# What do cells actually want?

**DOI:** 10.1186/s13059-016-0983-3

**Published:** 2016-05-23

**Authors:** Adam M. Feist, Bernhard O. Palsson

**Affiliations:** Department of Bioengineering, University of California, San Diego, La Jolla 92093-0412 USA

## Abstract

Genome-scale models require an objective function representing what an organism strives for. A method has been developed to infer this fundamental biological function from data.

Please see related Research article: www.dx.doi.org/10.1186/s13059-016-0968-2

## Evolutionary pressures on genome-scale science

Genome-scale models of metabolism appeared soon after the first genome sequences became available [1]. Metabolic models are built on three foundations:

1. *Reconstruction of the metabolic network in an organism of interest*. Reconstruction consists of building the specific metabolic reactome and linking it to its encoding genes.
2. *Recasting of this biochemical, genetic and genomic information into a mathematical network*. This model formulation process defines the constraints under which the network must operate.
3. *Formulation and application of an objective function to search through the network for the “best” solution to optimize the objective function*. Application of an objective function is often necessary as metabolic networks inherently have multiple degrees of freedom and can possess non-unique solutions.

Theodosius Dobzhansky famously wrote, “Nothing in biology makes sense except in the light of evolution” [2]. The objective function strives to formalize this statement mathematically. It is used to represent distal causation—that is, the change in organism function over many generations as its fitness improves. The objective function is thus the biologist’s function as it is not based on physical or chemical causation. In genome-scale models, laws describing physical and chemical causation are cast as constraints on organism function as they limit achievable homeostatic states. Knowing the objective function is thus a fundamental issue in biology.

## How then does one determine cellular desires?

It is a grand challenge to determine what actual objective functions are. Two approaches have been used to elucidate appropriate objective functions. The first one is hypothesis based. For instance, the hypothesis that cells optimize their growth rate in nutritionally complete medium has been fairly revealing in studies of optimal metabolism in microorganisms [3, 4]. In fact, optimization of growth rate has been put to a direct experimental test through adaptive laboratory evolution [5]. Such experiments have now been automated, opening up large-scale experimental studies of objective functions defined by the selection pressure imposed. However, these objective functions are “man-made.”

The second approach is to infer the objective function from observed cellular functions in their natural state. The initial constraint-based study of mammalian metabolism used experimental data to conclude that its observed state was most likely determined by minimizing formation of reactive oxygen species (ROS) [6]. This inverse approach to finding the objective function from data was subsequently formalized through optimization methods [7, 8].

## Using data to drive inference

In a study appearing in this issue, Danel Segré and colleagues further study the inference approach though the development of a method they call inverse flux-balance analysis (invFBA). Their goal is to address “…whether it is possible to use the flux balance framework to associate possible metabolic objective functions to a given measured set of genome-scale fluxes. In other words, we seek to understand whether it is possible to say that a given organism was optimized to favor some reactions at the expense of others” [9].

The authors first perform a computationally based test of their method. They generate optimal growth solutions for a genome-scale model of *Escherichia coli* growing on different carbon sources. They show that if these flux states are fed to invFBA, it infers the growth rate objective functions accurately. However, the solution to an inverse problem is not unique and alternative objective functions can generate the same flux map. Using an “objective variability analysis” to search the range of alternative objective functions, the authors show that maximizing the substrate uptake rate represents an equivalent objective, based on the fixed metabolic flux state given to invFBA.

Unlike computational solutions, actual data come with experimental error. The authors next examine the robustness of invFBA in the face of experimental error. They find that, as the magnitude of the noise increases, the performance of invFBA deteriorates, as expected, with a major downshift when the noise level is between 1 and 10 % relative to the flux norm.

The authors apply invFBA to two experimental situations: data generated in their lab for *Shewanella oneidensis* over a time-series, and *E. coli* strains evolved for over 50,000 generations. For the first case, they find that invFBA was able to capture trends in secretion and update of exchanged metabolite fluxes for pyruvate, glycolate, and acetate in *S. oneidensis*. Furthermore, it was determined that biomass production was the likely objective function during the experiment at the different time steps. For the second case, examining evolved cell lines, invFBA was used to understand the tradeoff between two specific “biologically interesting fluxes” from the evolved cell lines—growth versus respiration flux. The algorithm was able to link high-acetate-producing cell lines to a low respiratory objective, and vice versa, providing a link between correlated pathways for the observed phenotypes.

The majority of published studies use the growth rate (i.e., biomass) equation as an appropriate objective function. However, there are many situations of interest where the primary function of the cell being analyzed is not growth. Such situations include the homeostatic functions of human cells in tissues, the metabolic function of the mitochondria or chloroplasts, or microbial cells in a biofilm community. The invFBA approach might be able to analyze such circumstances and provide novel insights into their cellular physiology.

## The challenges that remain

The invFBA method represents progress towards the important goal of inferring objective functions from experimental data, although, as the authors state, significant challenges remain. Perhaps the major hurdle is to generate data sets large enough to fully implement invFBA. The ^13^C-labeled experiments that are used to measure experimental fluxes generate relatively few flux values, and, in fact, often reduced-stoichiometric models are used to compute some of these fluxes. Thus, further development of inverse methods might follow that make optimal use of the available fragmented experimental data. Together, such efforts move us closer to the goal of being able to determine objective functions based on experimentation and yield a realistic understanding of perhaps the most fundamental of all biological (mathematical) functions.

## References

[1] Edwards JS, Palsson BO (1999). Systems properties of the *Haemophilus influenzae* Rd metabolic genotype. J Biol Chem.

[2] Dobzhansky T (1973). Nothing in biology makes sense except in the light of evolution. Am Biol Teacher.

[3] Edwards JS, Ibarra RU, Palsson BO (2001). In silico predictions of *Escherichia coli* metabolic capabilities are consistent with experimental data. Nat Biotechnol.

[4] Schuetz R, Zamboni N, Zampieri M, Heinemann M, Sauer U (2012). Multidimensional optimality of microbial metabolism. Science.

[5] Ibarra RU, Edwards JS, Palsson BO (2002). *Escherichia coli* K-12 undergoes adaptive evolution to achieve in silico predicted optimal growth. Nature.

[6] Savinell JM, Palsson BO (1992). Optimal selection of metabolic fluxes for in vivo measurement. II. Application to *Escherichia coli* and hybridoma cell metabolism. J Theor Biol.

[7] Burgard AP, Maranas CD (2003). Optimization-based framework for inferring and testing hypothesized metabolic objective functions. Biotechnol Bioeng.

[8] Gianchandani EP, Oberhardt MA, Burgard AP, Maranas CD, Papin JA (2008). Predicting biological system objectives de novo from internal state measurements. BMC Bioinformatics.

[9] Zhao Q, Stettner A, Reznik E, Paschalidis I Ch, Segrè D. Mapping the landscape of metabolic goals of a cell. Genome Biol. 2016. doi:10.1186/s13059-016-0968-2. 10.1186/s13059-016-0968-2 PMC4878026 27215445

